# Nitric oxide maintains cell survival of *Trichomonas vaginalis* upon iron depletion

**DOI:** 10.1186/s13071-015-1000-5

**Published:** 2015-07-25

**Authors:** Wei-Hung Cheng, Kuo-Yang Huang, Po-Jung Huang, Jo-Hsuan Hsu, Yi-Kai Fang, Cheng-Hsun Chiu, Petrus Tang

**Affiliations:** Graduate Institute of Biomedical Sciences, College of Medicine, Chang Gung University, Kweishan, Taoyuan, Taiwan; Molecular Regulation and Bioinformatics Laboratory, Department of Parasitology, College of Medicine, Chang Gung University, Kweishan, Taoyuan, Taiwan; Molecular Medicine Research Center, Chang Gung University, Kweishan, Taoyuan, Taiwan; Bioinformatics Center, Chang Gung University, Kweishan, Taoyuan, Taiwan; Molecular Infectious Disease Research Center, Chang Gung Memorial Hospital, Kweishan, Taoyuan, Taiwan

**Keywords:** Iron-deficiency, Nitric oxide, Arginine, Ubiquitin-proteasome system, Hydrogenosomal membrane potential, Cell survival

## Abstract

**Background:**

Iron plays a pivotal role in the pathogenesis of *Trichomonas vaginalis*, the causative agent of highly prevalent human trichomoniasis. *T. vaginalis* resides in the vaginal region, where the iron concentration is constantly changing. Hence, *T. vaginalis* must adapt to variations in iron availability to establish and maintain an infection. The free radical signaling molecules reactive oxygen species (ROS) and reactive nitrogen species (RNS) have been proven to participate in iron deficiency in eukaryotes. However, little is known about the roles of these molecules in iron-deficient *T. vaginalis*.

**Methods:**

*T. vaginalis* cultured in iron-rich and -deficient conditions were collected for all experiments in this study. Next generation RNA sequencing was conducted to investigate the impact of iron on transcriptome of *T. vaginalis*. The cell viabilities were monitored after the trophozoites treated with the inhibitors of nitric oxide (NO) synthase (L-NG-monomethyl arginine, L-NMMA) and proteasome (MG132). Hydrogenosomal membrane potential was measured using JC-1 staining.

**Results:**

We demonstrated that NO rather than ROS accumulates in iron-deficient *T. vaginalis.* The level of NO was blocked by MG132 and L-NMMA, indicating that NO production is through a proteasome and arginine dependent pathway. We found that the inhibition of proteasome activity shortened the survival of iron-deficient cells compared with untreated iron-deficient cells. Surprisingly, the addition of arginine restored both NO level and the survival of proteasome-inhibited cells, suggesting that proteasome-derived NO is crucial for cell survival under iron-limited conditions. Additionally, NO maintains the hydrogenosomal membrane potential, a determinant for cell survival, emphasizing the cytoprotective effect of NO on iron-deficient *T. vaginalis*. Collectively, we determined that NO produced by the proteasome prolonged the survival of iron-deficient *T. vaginalis* via maintenance of the hydrogenosomal functions.

**Conclusion:**

The findings in this study provide a novel role of NO in adaptation to iron-deficient stress in *T. vaginalis* and shed light on a potential therapeutic strategy for trichomoniasis.

**Electronic supplementary material:**

The online version of this article (doi:10.1186/s13071-015-1000-5) contains supplementary material, which is available to authorized users.

## Background

*Trichomonas vaginalis* is a unicellular pathogen that causes human trichomoniasis, one of the most prevalent sexually transmitted diseases worldwide [1]. Iron deficiency in the host affects several biological processes in *T. vaginalis*, including cell proliferation, cytotoxicity and immune evasion [2–4]. Iron-containing proteins, such as lactoferrin and hemoglobin, can be used by *T. vaginalis* [5], and these proteins are mainly supplied by menstrual blood [6]. The constant change in environmental iron availability might be the major challenge for the protist to survive in the vaginal region [5]. Therefore, the protist must adapt to an environment with different iron concentrations to establish or maintain an infection. The iron level has to be tightly controlled because overload or deficiency can cause cellular damages [7, 8]. However, the mechanisms that help *T.vaginalis* cope with iron stresses remain poorly understood.

Redox homeostasis is an important issue for cellular functions because excessive free radicals destroy biomolecules [9]. A previous study demonstrated that superoxide dismutase (SOD) is required for *T. vaginalis* during the initial phase of oxygen stress [10]. The iron-containing SOD cannot perform its function normally under iron-deficient situations [11], implying that iron deficiency may induce oxidative stress. In addition to the damaging effects of free radicals, reactive oxygen species (ROS) and reactive nitrogen species (RNS) are also crucial for the signal transduction, that is responsible for the regulation of cellular processes and metabolic activities [12]. Therefore, these molecules might be beneficial for iron-deficient cells. To date, there have been no reports on intrinsic ROS or RNS production or the corresponding signaling pathways involved in iron-deficient *T. vaginalis*. Hence, we investigated the cellular events regulated by these multi-functional molecules.

Hydrogenosome, a mitochondrial homolog, is the center of energy metabolism as well as the iron-sulfur cluster assembly machinery [13]. Iron-sulfur cluster containing proteins are key molecules responsible for ATP production [14]. In an iron-deficient state, the hydrogenosomes are impaired, affecting the efficiency of energy generation [15]. Additionally, iron deficiency reduces hydrogenosomal membrane potential [16], which is a determinant for the health status of the trichomonad cells [17]. These evidences imply that iron deficiency might cause impairments in cells by disrupting the hydrogenosomal functions. In mammals, redox molecules are associated with the biogenesis and activity of mitochondria [18]. For instance, nitric oxide (NO) regulates the biogenesis of mitochondria via cGMP (cyclic guanosine monophosphate)-dependent signaling [19]. This suggests that redox regulators might also participate in the modulation of hydrogenosomal activity in *T. vaginalis*.

Until now, the detailed mechanisms modulating the survival of *T. vaginalis* in iron-deficient environments were unclear. In this study, we found that NO dramatically accumulated in iron-deficient *T. vaginalis*. More importantly, we determined that NO production is dependent on the ubiquitin-proteasome system (UPS) and arginine, and that the process maintains the hydrogenosomal membrane potential to enhance the viability of *T. vaginalis* in iron-deficient environments.

## Methods

### *T. vaginalis* culture and treatments

*T. vaginalis* ATCC strain 30236 was cultured at 37 °C in yeast extract, iron-serum (YI-S) medium containing 80 μM ferrous ammonium citrate (FAC, Sigma-Aldrich, USA) (iron-rich condition) [20]. Iron-deficient cells were grown in YI-S medium without iron supplementation and treated with 180 μM of the iron chelator dipyridyl (DIP, Sigma-Aldrich) at a cell density of 10^6^ cells/ml. The cells for assays were harvested from the mid-log phase of iron-rich cells and the iron-deficient cells were cultured with DIP for 6 h. The trypan blue exclusion assay was used to monitor the growth of cells.

NO synthase inhibitor NG-monomethyl L-arginine (L-NMMA, Sigma-Aldrich, 1 and 3 mM), proteasome inhibitor MG132 (Sigma-Aldrich, 5 and 10 μM), and arginine (Sigma-Aldrich, 5 mM) were also added in different experimental groups.

### Total RNA extraction

The total RNA of *T. vaginalis* cultured in iron-rich and -deficient medium was extracted as follows. The cell pellets (2 × 10^7^cells) were resuspended by adding 1 ml TRI Reagent (Life Technologies) and were incubated at room temperature for 5 min, followed by the addition of 200 μl chloroform and incubation at room temperature for 15 min. The RNA fraction was collected after 16,750 × g centrifugation at 4 °C for 15 min. Diethylpyrocarbonate (DEPC)-treated 70 % alcohol was used to wash the RNA pellets, and the dried RNA was reconstituted after adding the DEPC-treated water.

### Quantitative real-time PCR

The mRNA was reverse-transcribed to cDNA by reverse transcriptase reactions. The first step contained 5 μg total RNA, 50 nM RT primer (oligo-dT, Invitrogen, Life Technologies), and 0.25 mM dNTPs in 10 μl; the mixture was incubated at 65 °C for 5 min. cDNA Synthesis Mix (0.75 U/μl ThermoScript™ III reverse transcriptase (Invitrogen, Life Technologies), 0.2 U/μl RNase out (Promega, USA), and 0.05 M dithiothreitol (DTT, Sigma-Aldrich) was added to the mixture, and the RNA was converted to cDNA in a series of incubations (25, 50, and 85 °C for 5, 60 and 15 min, respectively). RNA was removed from the RNA-cDNA hybrids after treatment with RNase H for 20 min. All RNA samples were quantified using a UV Spectrophotometer SMA 1000 (Merinton, China) before experiments.

Real time PCR was performed to validate the expression levels of antioxidants as previously described [15, 21] (Additional file 1). Ribosomal protein L8 (TVAG_104490) was used as the internal control for data normalization (forward: TTGCGGTATCAAGATGAACCCAG, reverse: GAACCAAAGCTTTATGCAAGGTTGA). The following reagents composed the reaction mixture: 2X reaction mix (Ampliqon, Denmark), primers (0.5 μM), and 50 ng cDNA from each condition, with the volume adjusted to 20 μl with sterile water. The reaction was performed on an MX3000p (Stratagene, Agilent Technologies), using ROX dye as a reference and SYBR green as a quantifying signal [22]. The data were calculated by MX pro program version 4.10 (Stratagene, Agilent Technologies), representing the relative expression level of each gene in different conditions.

### Total antioxidant capacity test

The total antioxidant capacities of cells cultured in different iron concentrations were determined as described by the manufacturer (BioVision, USA). A total of 2 × 10^6^ cells were collected from each group and lysed by 100 μl Radio-ImmunoPrecipitation Assay (RIPA) lysis buffer. After centrifugation at 16,750 × g for 10 min, 15 μl of the samples were added and the volume was adjusted to 100 μl with the assay diluent. Working reagent (100 μl for each sample) was prepared and then combined with Cu^++^ and assay diluent in a ratio of 1:49. The 100 μl of diluted samples and trolox standards (0, 4, 8, 12, 16, and 20 nmol/ well) were mixed with 100 μl of working reagent and incubated at room temperature for 30 min. The absorbance at 570 nm of each sample was measured by using an ELISA reader, SpectraMax M2e (Molecular devices, USA). The values were expressed as mM of Cu^++^ reduced, converted from standard trolox equivalent.

### Reactive oxygen species (ROS) detection

ROS detection was performed using a previously described approach with slight modification [23]. Briefly, 5 × 10^6^ cells were collected from different cultivations. After wash steps, the cell pellets were treated with 1 ml pre-warmed phosphate buffered saline (PBS), and seeded 100 μl cells in the wells of the micro-plate. A final concentration of 1 μM chloromethyl-2′, 7′-dichloroflurescein diacetate (CM-DCFDA, Molecular Probes, Life Technologies) was added into each well, and incubated at 37 °C for 1 h. Fluorescence signals were detected using an ELISA reader (excitation/ emission = 490/525 nm).

### Intracellular nitric oxide (NO) detection

NO was detected by following the manufacturer’s procedures. A total of 5 × 10^6^ cells were collected from each culture system. After wash steps, cell pellets were resuspended with 1 ml PBS, and the 100 μl aliquots of cells were seeded into dark micro-centrifuge tubes containing 1 μl of 1 mM 4-amino-5-methylamino-2′, 7′-difluorofluorescein diacetate (DAF-FM DA, Molecular Probes, Life Technologies). After incubation at 37 °C for 1 h, the loading buffer was washed out, and the cell pellets were resuspended by the addition of 1 ml PBS and then incubated for 30 min. The fluorescent signals were detected with an ELISA reader (excitation/ emission as 495/ 520 nm) and the fluorescence intensities were recorded.

### Next generation sequencing (NGS) and data analysis

The NGS analysis was performed as previously described [24]. Briefly, the prepared cDNA (iron-rich and -deficient) were fragmented for library construction and sequenced using the Illumina sequencing platform (HiSeq™ 2000, Illumina, USA). The raw data sets were processed using CLC Genomic workbench (CLC bio) software (version 7). Reads in each gene set were identified with the tool “Mapping to reference” against the G3 reference genome (TrichDB, release-1.3) [25]. All parameters were setup as the recommended defaults. Besides, the length fractions and similarity were changed to 0.9 and 0.8, respectively. Reads Per Kilobase per Million mapped reads (RPKM) of each gene were calculated and used as the normalized gene expression level (Additional file 2). The basic information generated from this analysis is shown in Additional file 3.

### Proteasome activity assay

Proteasome activities of different iron availabilities were determined by a fluorescent approach (proteasome activity fluorometric assay kit, BioVision) following the manufacturer’s protocol. The iron-rich and -deficient cultured cells (2 × 10^6^) were lysed in 100 μl of 0.5 % NP-40 (Sigma-Aldrich). The reaction mixture contained 10 μl of testing samples, 90 μl assay buffer, and 1 μl of proteasome substrate in the wells of the micro-plate. MG132 (proteasome inhibitor) treated group was used as the negative control for calibration. The fluorescence signal can be detected when the substrate is digested by proteasome in cell lysates. After 10 and 30 min of incubation at 37 °C, the values of experimental groups as well as the aminomethylcoumarin (AMC) standards (0, 20, 40, 60, 80, and 100 pmol/well) were measured simultaneously by using an ELISA reader (excitation/emission = 350/440 nm). The kinetics of the proteasome activity was generated after subtracting fluorescence intensities detected from 2 time points of each sample. The values were expressed in nmol/min/ml.

### Hydrogenosomal membrane potential measurement

The hydrogenosomal membrane potential was measured as previously described [26]. We harvested 1 × 10^6^ cells from each of the treatments (DIP only, DIP plus MG132, DIP plus MG132 and arginine) after 6 h of drug exposure. The cell pellets were resuspended in 1 ml PBS. No stained cell (blank) without JC-1 (Molecular Probes, Life Technologies) treatment was prepared for flow cytometry adjustment. All testing cells included a negative control (treated with mitochondrial membrane potential disrupter carbonyl cyanide 3-chlorophenyl hydrazone (CCCP) in 50 μM) were treated with 2 μM JC-1, incubating at 37 °C for 30 min. The cells were washed once with PBS, and the cell was then resuspended with 0.5 ml PBS. The red fluorescence intensities alternations in experimental groups were detected by flow cytometry analysis.

### Statistical analysis

Student’s t-tests were performed on the quantified data derived from biological assays, using GraphPad Prism 5 software. Asterisks were used to represent the significance of each assay as determined through the p value (*: *p* < 0.05; **: *p* < 0.01; ***: *p* < 0.001).

## Results

### *T. vaginalis* survives in iron-deficient condition

**Fig. 1:**
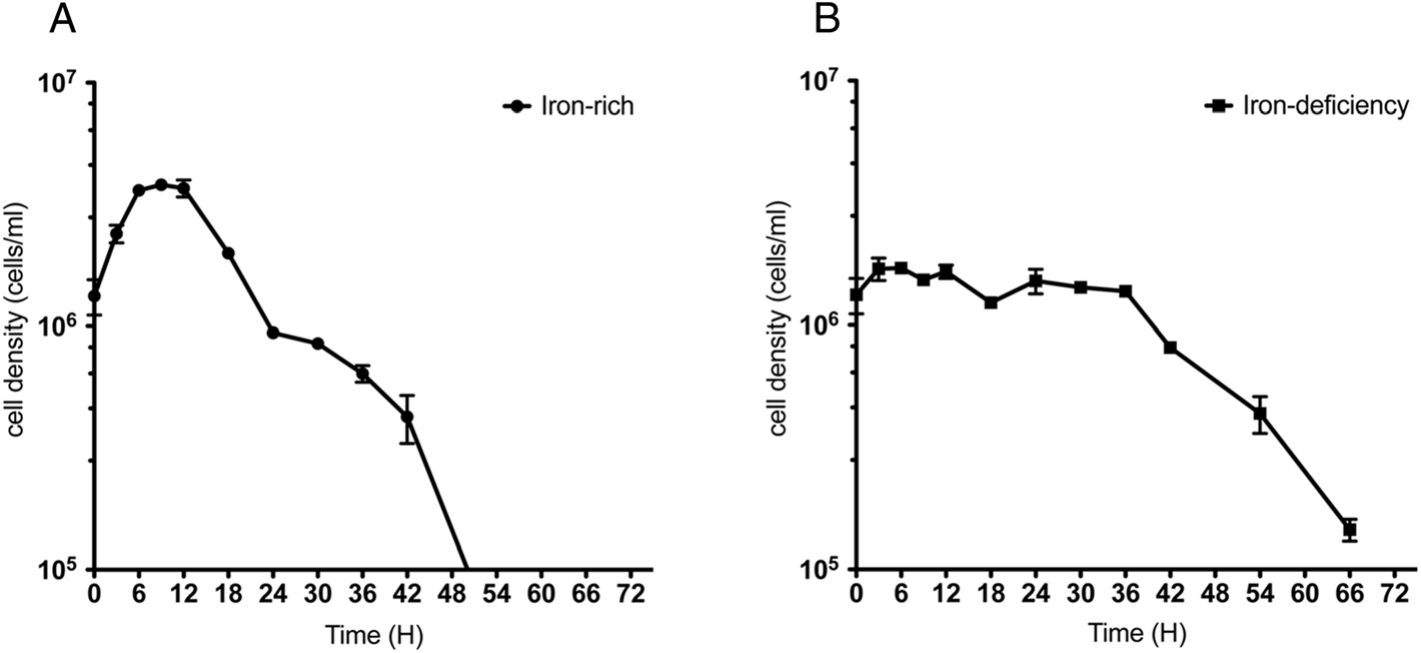
*T. vaginalis* extends the survival time when cultured under iron-deficient conditions. The growth of cells cultured in iron-rich (IR, 80 μM FAC, **a**) and -deficient (ID, 180 μM DIP, **b**) media. The number of viable cells was monitored every three hours using the trypan blue exclusion assay. The initial cell density is 1 × 10^6^cells/ ml. The data are presented as the mean ± SD of three independent experiments

It is known that iron is essential for cell proliferation in *T. vaginalis*. Iron restriction causes an increase in doubling time and a decrease in the maximum cell density [27]. Until now, no report indicates the viability of *T. vaginalis* upon iron deficiency. To monitor the cell viability under iron-deficient condition, we cultured the cells with a higher initial cell density (10^6^ cells/ml) in the medium containing 180 μM DIP [24, 28]. The iron-rich cells reached the maximum cell density of approximately 3.5 × 10^6^ (cells/ml) at 6 h after inoculation, followed by a rapid decline (Fig. 1a). The viable cells were reduced to 1 × 10^5^ (cells/ml) at approximately 50 h in iron-rich cultivation. In contrast, we found that the maximum cell density of iron-deficient cells was 2-fold (~1.5 × 10^6^ cells/ml) less than that of iron-rich cells (Fig. 1b). Interestingly, our result showed that the survival of iron-deficient cells was further extended to 66 h with a cell density of 1 × 10^5^ viable cells per ml. This phenomenon reflects that although iron deficiency affects cell proliferation, *T. vaginalis* is capable of adapting to an iron-deficient environment and survives for a longer period. However, the underlying mechanism responsible for cell survival during iron deficiency is still unclear, which might be an important issue in elucidating how *T. vaginalis* establishes and maintains infection in the vaginal region.

### NO is accumulated in iron-deficient *T. vaginalis*

**Fig. 2:**
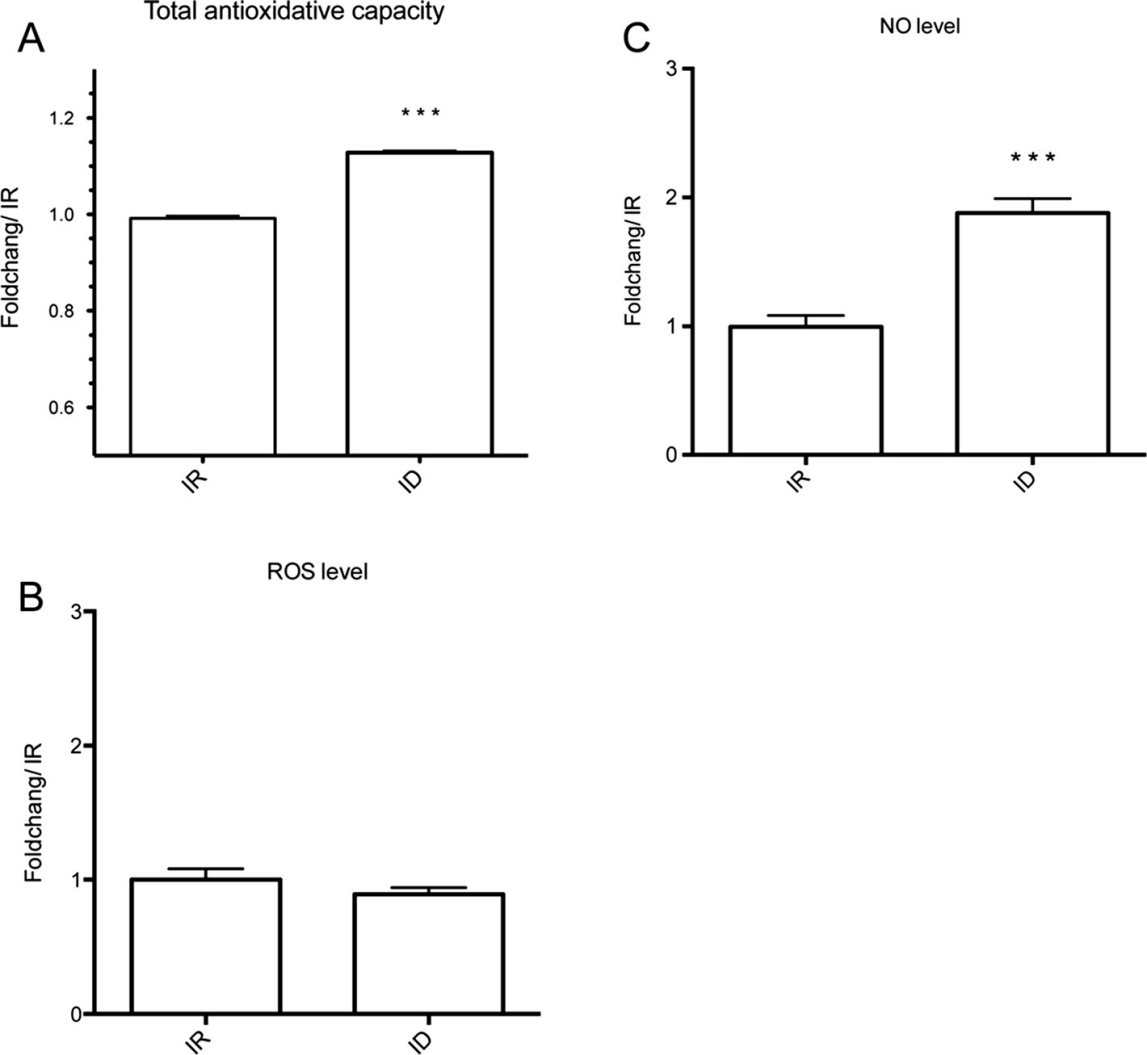
NO accumulated in iron-deficient *T. vaginalis.* **a** Antioxidant capacity of iron-rich (IR) and -deficient (ID) cells representing the reducing power (Cu^++^ to Cu^+^) of each lysate. ROS (CM-DCF DA) **b** and NO (CM-DAF FM) **c** were examined. IR, iron rich (80 μM FAC); ID, iron deficient (180 μM DIP). The data are presented as the mean ± SD of three independent experiments. *** *p* < 0.001, compared with the IR group

Previous studies have indicated that iron-deficient cells exhibit up- and down-regulation of thiol- and iron-dependent antioxidant defense systems, respectively [15, 21]. We confirmed the expression patterns of the iron-dependent antioxidants SOD (TVAG_039980, TVAG_120340) and rubrerythrin (TVAG_064490, TVAG_275660) and the thiol-dependent thioredoxin peroxidase (TVAG_114310, TVAG_455310) in cells cultured under iron-rich and -deficient conditions by using quantitative RT-PCR. The results revealed a trend similar to previous studies (Additional file 4). Furthermore, we examined the cellular reducing power in iron-rich and -deficient cells by measuring the amount of copper reduction (Cu^++^ to Cu^+^), which is used as a general indicator for antioxidant capacity. As shown in Fig. 2a, there is a significant increase in copper reduction in the iron-deficient group compared with iron-rich group. Together with quantitative RT-PCR analysis of multiple antioxidant genes, these data confirm that the antioxidative response of iron-deficient cells is stronger than that of iron-rich cells.

To understand whether the redox molecules increased simultaneously with antioxidants upon iron deficiency, we used individual fluorescence indicators to detect ROS and RNS (NO). As shown in Fig. 2b, there is no significant difference in ROS production between iron-rich and -deficient groups. Surprisingly, the amount of NO is elevated when the environmental iron concentration decreased (Fig. 2c), with an approximate 2-fold increase in iron-deficient conditions. These data suggest that the increase of intracellular NO production and the antioxidants for redox maintenance in *T. vaginalis* occur under iron-deficient conditions. The accumulated NO and the corresponding signaling pathways were likely crucial for iron-deficient cells; however, limited information is known about the relationship of NO and iron deficiency in *T. vaginalis*.

### Transcriptomics analysis revealed NO-related responses in iron-restricted *T. vaginalis*

Previous studies utilizing NGS-based transcriptomics analysis in *T. vaginalis* provided insights into the detailed mechanical information [10, 24]. Therefore, this technique creates the opportunity to identify the novel genes or pathways in *T. vaginalis* associated with iron deficiency. To date, little is known about the biological functions of intrinsic NO in *T. vaginalis*, and the massive gene families make it difficult to discern the participating genes. We performed an NGS-based transcriptomics analysis of the iron-deficient cells compared with iron-rich cells to monitor the NO-related events. A total of 51,519,438 and 53,444,250 high quality reads (100 bases in length) were generated from the iron-rich and -deficient cDNA libraries, respectively (Additional file 3). These data sets covered approximately 100-fold of the *T. vaginalis* genome that contains more than 60,000 genes. After mapping the reads to the *T. vaginalis* genome, there were 28,256 and 33,976 protein-coding genes expressed in iron-rich and -deficient cells, respectively (Additional file 3). Table 1 showed the most significantly up-regulated genes in iron-deficient cells (RPKM > 1000 and 10-fold higher than that in iron-rich cells), including metabolic enzymes, proteolysis, oxidoreductases, and hypothetical genes. Among the highly expressed genes, hydroxylamine reductase (TVAG_336320), carboxymuconolactone decarboxylases (TVAG_107080, 256720), and regulators of cell morphogenesis and NO signaling (TVAG_167830) are all related to NO signaling pathways in other organisms [29–31]. The expression patterns of these genes were also validated by quantitative RT-PCR (Additional file 5). This further prompted us to study the roles of NO in iron-deficient *T. vaginali*s.

**Table 1:** The most expressed genes in iron-deficient *T. vaginalis*

GeneID	TrichDB Annotation	RPKM (IR)	RPKM (ID)
METABOLIC ENZYMES			
TVAG_171090	Malate dehydrogenase, putative	1171.96	19,482.82
TVAG_344880	Alcohol dehydrogenase, putative	115.8	3,902.75
TVAG_038440	Fructose-bisphosphate aldolase, putative	63.12	1,715.45
TVAG_196700	Glutamate dehydrogenase, putative	41	1,787.34
OXIDOREDUCTASES			
TVAG_167830	Regulator of cell morphogenesis and NO signaling (RCMNS) (metal ion binding)	237.68	2,776.24
TVAG_107080	4-carboxymuconolactone decarboxylase 2	11.21	3,030.84
TVAG_256720	4-carboxymuconolactone decarboxylase 1	2.82	3,363.03
TVAG_336320	Hydroxylamine reductase, putative	70.86	1089.89
PROTEOLYTIC PROCESSES			
TVAG_184150	Ubiquitin, putative	113.42	1563.65
TVAG_476160	Clan MG, family M24, aminopeptidase P-like metallopeptidase	77.07	1534.83
TVAG_386080	Clan MG, family M24, aminopeptidase P-like metallopeptidase	129.25	7169.21
OTHERS			
TVAG_469020	Biotin synthase, putative	4.15	1,508.21
TVAG_321740	Conserved hypothetical protein	67.62	8933.47
TVAG_059980	Conserved hypothetical protein	102.07	1,355.29
TVAG_307440	Conserved hypothetical protein	7.34	1,956.57
TVAG_491130	Conserved hypothetical protein	75.06	2,060.76
TVAG_488900	Conserved hypothetical protein	1.48	2,220.44

The genes listed in this table are only highly expressed (RPKM > 1000, fold-change > 10) in iron-deficient (ID) cells compared to iron-rich (IR) cells. RPKM, reads per kilobase per million mapped reads

### NO production is via arginine- and proteasome-dependent pathway

**Fig. 3:**
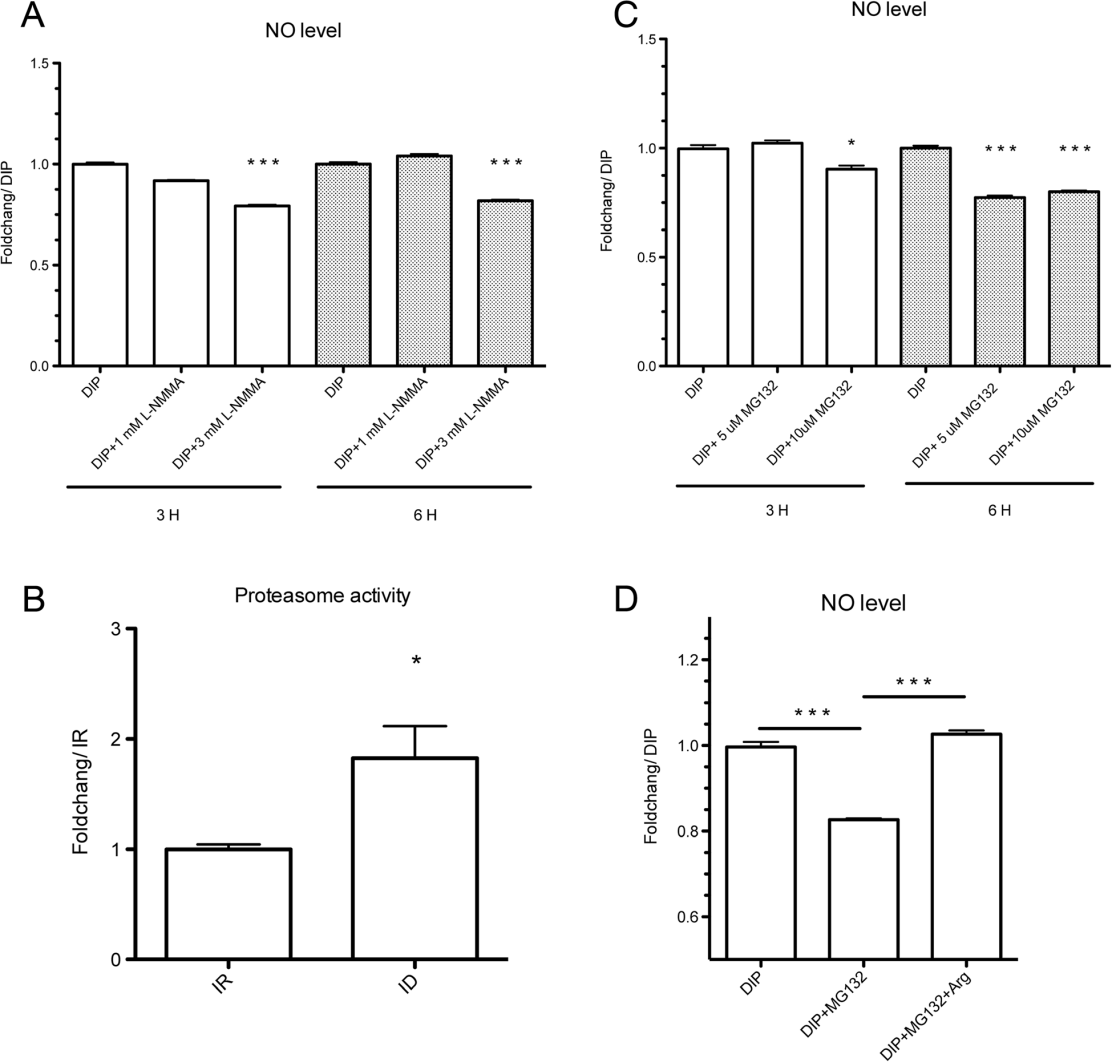
NO production is an arginine- and proteasome-dependent process. **a** NO levels in DIP (180 μM) and L-NMMA (1 and 3 mM) co-treated cells after 3 (white bars) and 6 h (gray bars) incubation. *** *p* < 0.001, compared with the DIP-only group. **b** Proteasome activities were measured in iron-rich (IR) and -deficient (ID) cultured cells by detecting the fluorescence signal intensity. * *p* < 0.05, compared with the IR group. **c** NO levels of DIP- and MG132-treated (1 and 3 μM) cells after 3 and 6 h of incubation. * (*p* < 0.05) and*** (*p* < 0.001), compared with the DIP-only group. **d** Co-treatment with MG132 and arginine (5 mM) in iron-deficient cells after 6 h of incubation.*** *p* < 0.001, based on fold-change between the indicated groups

It has been suggested that *T. vaginalis* probably uses arginine deiminase (ADI), with no putative nitric oxide synthase encoded, to produce NO when the culture is supplemented with arginine in aerobic conditions [32]. According to our transcriptomics data, ADIs (TVAG_344520, TVAG_467820, and TVAG_183850) exhibited up-regulation in iron-deficient conditions (Additional file 2). To understand whether arginine is the substrate for NO production, we treated the iron-deficient cells with 1 and 3 mM arginine derivative L-NG-monomethyl arginine (L-NMMA) and monitored the NO levels. As shown in Fig. 3a, the NO level in iron-deficient cells treated with L-NMMA decreased in a dose-dependent manner after 3 h of treatment, with an approximately 20 % reduction in NO in cells treated with 3 mM L-NMMA compared with the untreated group, suggesting the importance of arginine for NO production in iron-deficient *T. vaginalis*. The effect of L-NMMA on NO reduction could not be sustained for a long period in iron-deficient cells (Additional file 6), suggesting that either more arginine is catalyzed for NO production or L-NMMA is consumed at later time points.

The ubiquitin-proteasome system (UPS) is thought to be the machinery for maintaining an intracellular arginine pool for NO production [33]. Hence, we investigated whether the UPS plays a key role in NO production in *T. vaginalis* during iron deficiency. Indeed, the proteasome activity is significantly increased in iron-deficient cells (Fig. 3b). To elucidate the correlation between proteolysis and NO production, we detected the NO level in iron-deficient cells treated with or without the proteasome inhibitor, MG132. The data revealed that the cells treated with MG132 have a decrease of 20 % in NO production in a time- and dose-dependent manner (Fig. 3c). NO reduction in the MG132-treated group can be restored by the addition of arginine (Fig. 3d), confirming the function of UPS in arginine pool maintenance and NO production in iron-deficient *T. vaginalis*.

Because the change in the amount of NO in MG132-treated cells can be totally reversed by arginine, and there is a longer inhibitory effect of MG132 on NO production compared with L-NMMA treated cells (Additional file 6), we thereby used MG132 to deplete NO production and clarify the roles of NO in iron-deficient cells. Taken together, we demonstrated that NO production depends on arginine and the UPS activity in *T. vaginalis* under iron-deficient conditions.

### NO maintains cell survival in iron-deficient *T. vaginalis*

**Fig. 4:**
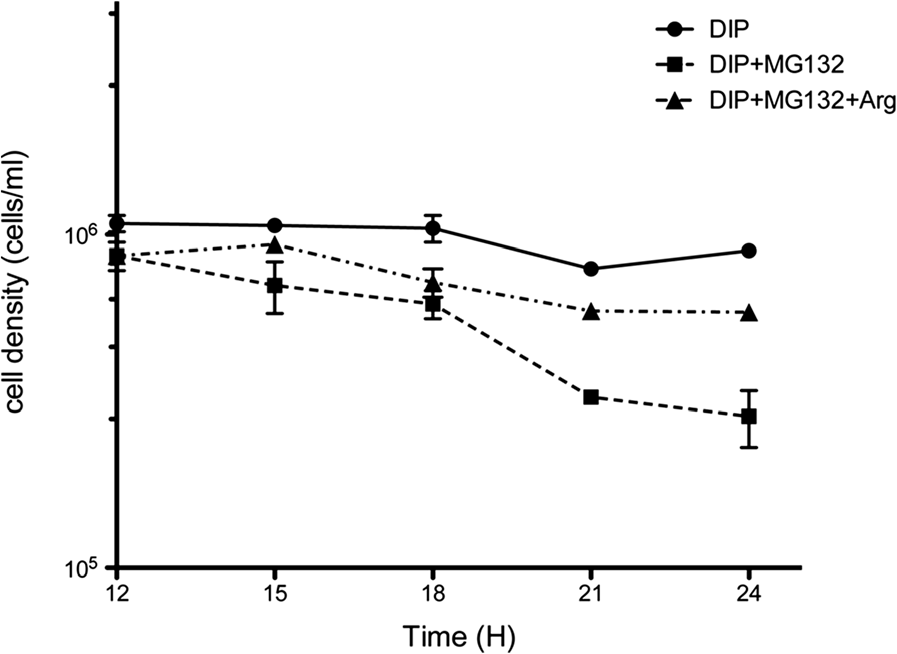
NO is important for cell survival in iron-deficient *T. vaginalis.* The growth of iron-deficient cells after treatment with different drugs was monitored every three hours using the trypan blue exclusion assay. The solid curve (circle) reveals the cell density of iron-deficient (DIP, 180 μM) cells; the dashed curve (square) reveals the cell density of MG132 (10 μM)-treated iron-deficient cells; the dot-dashed curve (triangle) reveals the cell density of MG132 and arginine (5 mM) co-treated iron-deficient cells. The data are presented as the mean ± SD of three independent experiments

To verify the role of NO in the survival of *T. vaginalis* under iron-deficient conditions, we monitored the growth of cells treated with or without MG132 by the trypan blue exclusion assay. As shown in Fig. 4, the viability of iron-deficient cells can be maintained at least 24 h after DIP treatment with a cell density of ~ 1 × 10^6^(cells/ml), whereas combined treatment with MG132 and DIP reduced the viable cells to ~3 × 10^5^(cells/ml) at 21h. Interestingly, the number of viable cells recovered by approximately 2-fold at 21h when the iron-deficient cells were co-treated with MG132 and arginine compared with MG132 treatment alone, suggesting that UPS-generated NO is important for cell survival. The UPS-inhibited cells could be rescued by the addition of single amino acid arginine, emphasizing the crucial function of UPS in NO production in iron-deficient *T. vaginalis*. In other words, this result demonstrates that NO is a pivotal molecule for cell survival when *T. vaginalis* resides in iron-deficient environments.

### NO enhances hydrogenosomal membrane potential in *T. vaginalis* upon iron deficiency

**Fig. 5:**
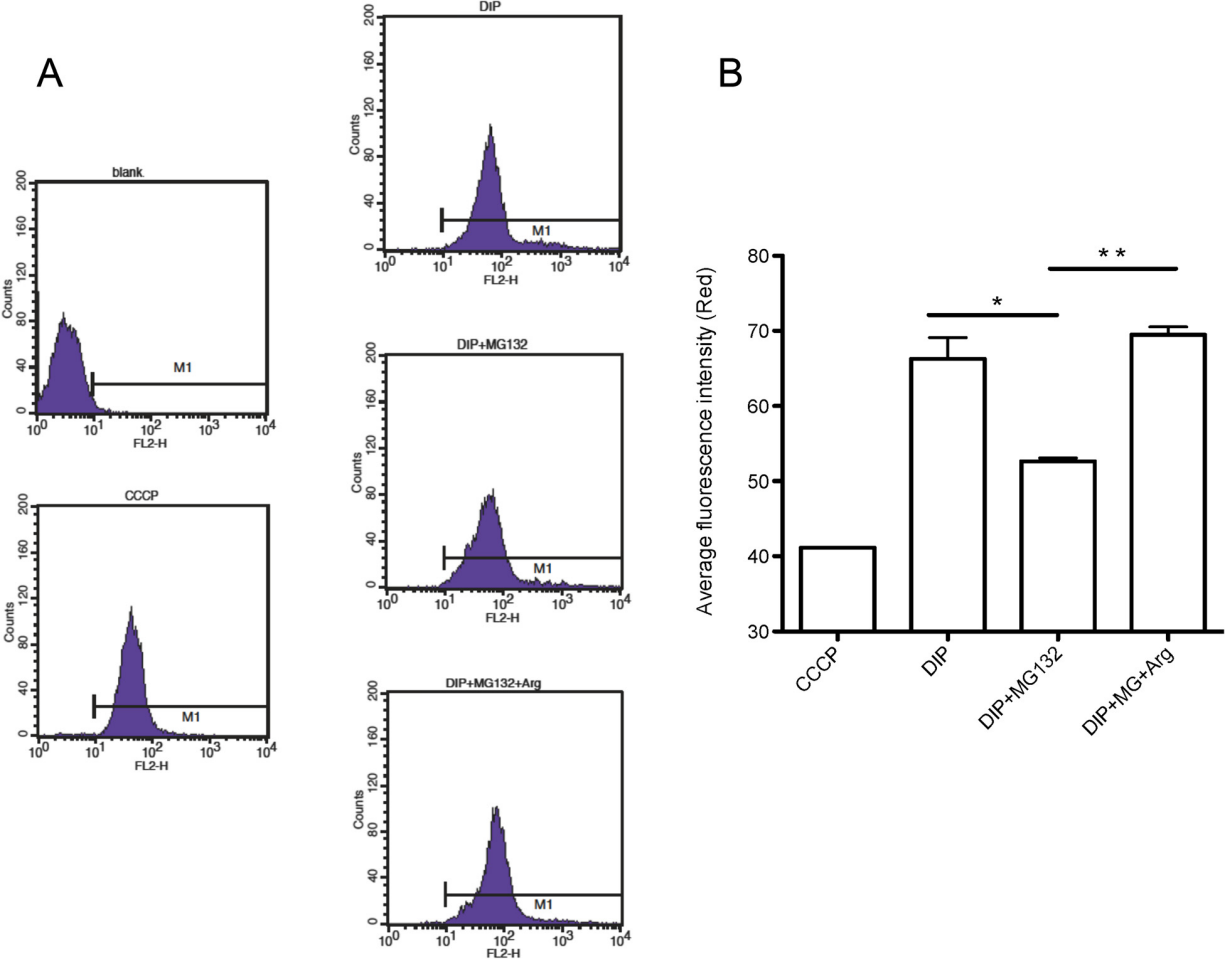
NO maintains the hydrogenosomal membrane potential of iron-deficient *T. vaginalis.* **a** The histograms indicate the red signal of the cells treated with DIP (180 μM), DIP-MG132 (10 μM), and DIP-MG132-arginine (5 mM). Red fluorescence represents a hydrogenosome with high membrane potential. The red fluorescence intensities of negative control group (CCCP treated cells, 50 μM) and experimental groups were detected by using flow cytometry after JC-1 staining. **b** Quantification data of the red fluorescence intensities (geometric mean) in panel **a** are shown. All tested groups were collected 6 h after drug treatments, and the data are presented as the mean ± SD of three independent experiments. *(*p* < 0.05) and **(*p* < 0.01), based on the differences between the indicated groups

Previous studies have proven that NO activates cGMP signaling to modulate mitochondrial biogenesis and activity in mammals [34]. Mitochondrial membrane potential is believed to be a determinant of metabolic activity and health status of a cell [35]. We utilized a cell-permeable dye JC-1 to monitor the hydrogenosomal membrane potential of *T. vaginalis*, which reflects the hydrogenosomal functions [17, 26]. Using JC-1 staining, we monitored the fluctuations in the red fluorescence-containing cells, which possessed hydrogenosomes with high membrane potential, in different iron concentrations. We used the cells treated with CCCP, a disruptor of mitochondrial membrane potential, as a negative control. As shown in Fig. 5a and b, the fluorescence intensities were reduced in the CCCP-treated cells, indicating that the measurement is suitable for analysis of hydrogenosomal membrane potential. We found that the red fluorescence intensity in MG132 treated iron-deficient cells was decreased significantly (*p* < 0.01) compared with untreated cells (Fig. 5b). Interestingly, the MG132-mediated reduction in red signal can be recovered by the addition of arginine (Fig. 5b), suggesting that NO maintains the hydrogenosomal membrane potential in iron-deficient *T. vaginalis*. These observations indicate that the hydrogenosomal membrane potential of MG132-treated cells can be reversed after co-treatment with arginine, confirming that NO functions in hydrogenosomal membrane potential maintenance. These data demonstrate that NO serves as a “keeper” for maintaining the functions of the hydrogenosome, which is positively correlated with prolonged survival in iron-deficient *T. vaginalis*.

## Discussion

*T. vaginalis* has been shown to adapt to environmental changes in the vaginal region, such as metabolic reprogramming and autophagy in response to glucose restriction [24]. Similarly, the shift of the energy production pathway from hydrogenosome to glycolysis in iron-deficient conditions has been demonstrated [15, 36] (Table 1). In the present work, we found that *T. vaginalis* can survive for an extended time in the environment without sufficient iron supplementation. Nevertheless, the mechanisms involved in the adaptation in iron-deficient situations are still largely unknown.

The up-regulation of NO in parallel with elevated antioxidant capacity suggests that NO signaling might be critical for iron-deficient *T. vaginalis* [37]. NO serves as a regulator to protect the cells from iron shortage-mediated damages [38]. For instance, accumulated NO functions in adjusting iron utilization and preventing oxidative stresses in iron-restricted plants [39, 40]. NO accelerates the release of iron from ferritin to re-balance the free iron concentration in iron deficient anemia models [41].

NO production from arginine-dependent pathway has been suggested in a previous study [32]. Nevertheless, it is still unknown how NO produced in *T. vaginalis*. In our work, we determined that *T. vaginalis* used arginine as the substrate for NO production under iron-deficient conditions without additional arginine supplementation. UPS, proteolytic machinery that commonly exists in eukaryotic and prokaryotic cells, functions in protein turnover and quality control [42]. The conserved ubiquitin-conjugating system and proteasome subunits can be found in the genome of *T. vaginalis*. However, none of them have been characterized. The UPS generates amino acid for cellular functions that have been examined in mammals, such as cysteine and arginine [33, 43]. We illustrated that *T. vaginalis* utilizes UPS-derived arginine for NO production under iron-deficient conditions. We also found that the UPS plays a pivotal role in the survival of iron-deficient cells, which correlated with NO production. This finding suggests that the functional role of UPS in NO production could be conserved in *T. vaginalis.*

Previous study suggests that *T. vaginalis* can also generate energy from arginine provided by either UPS or exogenous arginine via the arginine dihydrolase pathway [44]. However, according to our transcriptomics analysis, the compensatory responses were raised for energy production due to compromised hydrogenosomal energy metabolism of iron-deficient cells, such as carbohydrate metabolism (Table 1). In addition, the arginine dihydrolase pathway only contributes 10 % of ATP that generated from glycolysis [44]. Thus, we believe that exogenous arginine may trigger NO-related responses rather than ATP production to protect *T. vaginalis* from iron deficiency.

NO is a second messenger that controls cellular functions via triggering the production of cGMP [45]. The cGMP-regulated pathways are likely the important downstream effect of accumulated NO. We have monitored the cGMP levels in iron-deficient cells, which are 10 % greater than iron-rich cells (data not shown). The increased cGMP directly activates cGMP-dependent protein kinase (known as protein kinase G or PKG), which participates in signaling related to cell survival [46]. Among the 144 members of the protein kinase AGC family in the genome of *T. vaginalis*, no specific PKG was annotated [25]. It is difficult to pinpoint which gene is responsible for the cGMP-dependent reactions because there is high sequence similarity within the catalytic domain. Additionally, PKG is the potential drug target for *Toxoplasma* and *Plasmodium spp.* treatment [47, 48]. Therefore, the identification and characterization of PKG related to NO metabolism in *T. vaginalis*, especially under iron deficiency, is a crucial task in the future.

The NO-dependent increase in cGMP also regulates mitochondrial biogenesis in mammals [19, 45]. Previous studies demonstrated that iron-deficiency leads to a reduction in hydrogenosomal activity [16]. Here, we found that if NO production in iron-deficient cells is reduced, the membrane potential of hydrogenosome is further decreased. This suggests that NO is crucial for the functional maintenance of iron-restricted hydrogenosome. The mitochondrial membrane potential is a determinant for cell health [35]. Likewise, the linkage between hydrogenosomal membrane potential and cell death in *T. vaginalis* has been determined [17]. In fact, low mitochondrial membrane potential represents increased permeability, which is correlated with apoptosis in mammalian cells [35]. Accordingly, we proposed that NO-enhanced hydrogenosomal membrane potential is critical for cell survival under iron-deficiency.

## Conclusions

**Fig. 6:**
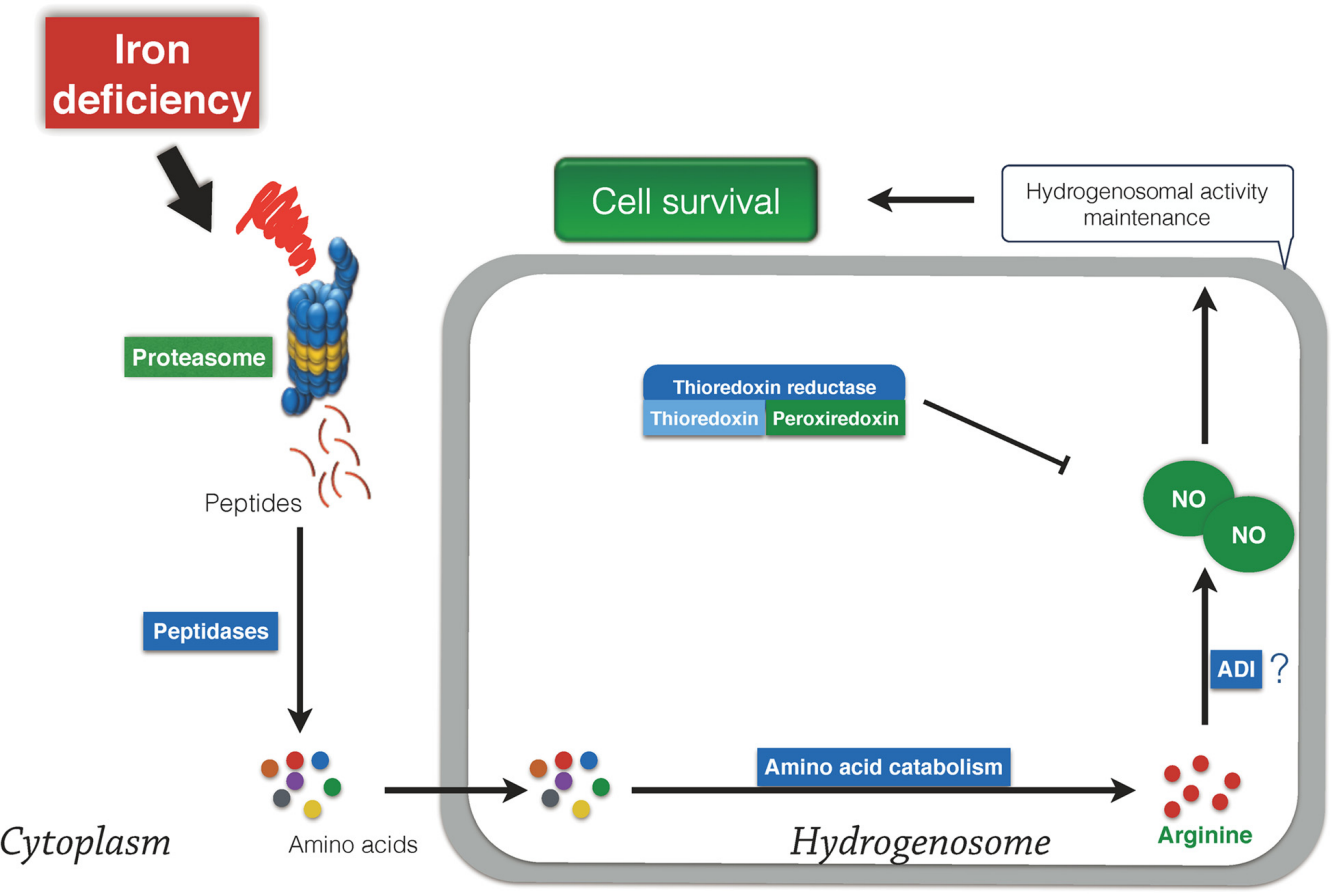
Scheme of the proposed model. In environments without sufficient iron availability, *T. vaginalis* activates the ubiquitin-proteasome system (UPS) to digest proteins and generate the amino acid arginine. It is likely that ADI, the enzyme with NO synthase activity in the hydrogenosome, utilizes arginine as the source for NO production [14, 32]. NO is crucial for the maintenance of hydrogenosomal function as well as antioxidant capacity. Both mechanisms protect *T. vaginalis* from iron deficiency-induced damage and prolong the survival of this parasite

In conclusion, we demonstrate, for the first time, that *T. vaginalis* utilized a NO-dependent regulatory network to survive in iron-deficient situations (Fig. 6). Once *T. vaginalis* encounters iron-limited environments, the protist generates more NO via proteasome-dependent pathway. The UPS-derived arginine is the substrate for NO production [33]. The generation of NO possibly takes place in the hydrogenosome since ADI, the enzyme with NO synthase activity, is found in this organelle [14, 32]. NO is a pivotal factor that modulates the hydrogenosomal membrane potential to protect cells from death. These mechanisms are vital for *T. vaginalis* to adapt to the continuous alternation of iron in the vaginal region, which is beneficial for establishment of an infection and parasitization.

## Additional files

Additional file 1:
**Primer sets for quantitative RT-PCR.**
Additional file 2
**Transcriptomic analysis of** ***T. vaginalis*** **under different iron concentrations by RNA-seq.**
Additional file 3
**Summary of NGS analysis.**
Additional file 4:
**Iron-dependent expression of antioxidants in** ***T. vaginalis*** **.** The expression levels of antioxidative defense systems in cells cultured under different iron concentrations were determined by using quantitative RT-PCR. IR, iron rich (80 μM FAC); ID, iron deficiency (180 μM DIP). SOD, superoxide dismutase; Rbr, rubrerythrin; TrxP, thioredoxin peroxidase. Additional file 5:
**Validation of NO-related genes identified from transcriptomics analysis. **The expression levels of iron deficiency-induced genes were verified by using quantitative RT-PCR. IR, iron rich (80 μM FAC); ID, iron deficiency (180 μM DIP). CMD, 4-carboxymuconolactone decarboxylase; HCP, hybrid cluster protein or hydroxylamine reductase; RCMNS, regulator of cell morphogenesis and NO signaling. Additional file 6:
**NO levels in iron-deficient** ***T. vaginalis*** **treated with NOS and proteasome inhibitors.** NO levels in iron-deficient cells treated with the NOS inhibitor (L-NMMA, 3 mM) and the proteasome inhibitor (MG132, 10 μM) for 12 h.

## Abbreviations

ROS: Reactive oxygen species
RNS: Reactive nitrogen species
NO: Nitric oxide
cGMP: cyclic guanosine monophosphate
L-NMMA: L-NG-monomethyl arginine
SOD: Superoxide dismutase
UPS: Ubiquitin-proteasome system
YI-S medium: Yeast extract, iron-serum medium
DIP: Dipyridyl
DEPC: Diethylpyrocarbonate
dT: deoxythymidine
dNTP: deoxynucleotide
cDNA: complementary DNA
RNase: Ribonuclease
DTT: Dithiothreitol
RT-PCR: Real-time polymerase chain reaction
PBS: Phosphate buffered saline
CM-DCF DA: Chloromethyl-2′, 7′-dichloroflurescein diacetate
ELISA: Enzyme-linked immunosorbent assay
DAF-FM DA: 4-amino-5- methylamino-2′, 7′-difluorofluorescein diacetate
NGS: Next generation sequencing
RPKM: Reads per kilobase per million mapped reads
AMC: Aminomethylcoumarin
CCCP: Carbonyl cyanide 3-chlorophenyl hydrazine
Rbr: Rubrerythrin
TrxP: Thioredoxin peroxidase
ADI: Arginine deiminase
