# Supplementary material for: Nitric oxide maintains cell survival of Trichomonas vaginalis upon iron depletion
Source: Parasit Vectors. 2015 Jul 25;8:393. doi: 10.1186/s13071-015-1000-5 (PMC4513698; doi:10.1186/s13071-015-1000-5)
Supplement: Additional file 1: — Primer sets for quantitative RT-PCR. [file 13071_2015_1000_MOESM1_ESM.pdf]

Additional file 1. Primer sets for quantitative RT-PCR

Oligo Name	Sequence
SOD-F (TVAG_039980)	TTAACACAGCACGCTGTCGAG
SOD-R (TVAG_039980)	AACATCAACCTTTTCTGGTGT
SOD-F (TVAG_120340)	CCTTACACAGCATGCTGTTGAGA
SOD-R (TVAG_120340)	CTCGACAGAGCCGAATTCCTTTGT
Rbr-F (TVAG_064490)	GAAGTTGCCAAGAAGCAAGG
Rbr-R(TVAG_064490)	CAGCAACATCAGCGAAGTGT
Rbr-F (TVAG_275660)	GAAGTCGCCAAGAAACAAGG
Rbr-R (TVAG_275660)	TTCACCAGCTGCAGCATTAC
TrxP-F (TVAG_455310)	ACTTGGGTGCGAGGTTCTTGGC
TrxP-R (TVAG_455310)	TTGGACATGACGAATTATTCCTTGA
TrxP-F (TVAG_114310)	GCGCTAAGATCGCTAAGGAA
TrxP-R (TVAG_114310)	AGCGAACTGGTAAGCCTTGA
CMD1-F (TVAG_256720)	GAGTTACAAAGCGACGGA
CMD1-R (TVAG_256720)	TCCAAAGACATTTCTAAAATGATT
CMD2-F (TVAG_107080)	TTAGGTCATGGAAAATGGGA
CMD2-R (TVAG_107080)	CATTCCCTTAGCAAATTTCA
HCP-F (TVAG_336320)	CAG TGC AAT GAC TCA TAC TCC C
HCP-R (TVAG_336320)	CCTTGATGCCGAAGTTCTTGA
RCMNS-F (TVAG_167830)	AGCCAGAAGTCAAATGGTATC
RCMNS-R (TVAG_167830)	TCTAGGTCATTGAGAGAGTTGAG

SOD, superoxide dismutase; Rbr, rubrerythrin; TrxP, thioredoxin peroxidase; CMD, 4- carboxymuconolactone decarboxylase; HCP, hybrid cluster protein or hydroxylamine reductase; RCMNS, regulator of cell morphogenesis and NO signaling.
