# Supplementary material for: Nitric oxide maintains cell survival of Trichomonas vaginalis upon iron depletion
Source: Parasit Vectors. 2015 Jul 25;8:393. doi: 10.1186/s13071-015-1000-5 (PMC4513698; doi:10.1186/s13071-015-1000-5)
Supplement: Additional file 3 — Summary of NGS analysis. [file 13071_2015_1000_MOESM3_ESM.pdf]

**Additional file 3. Summary of NGS analysis**

| Sample type | Total reads | Reads mapped to the reference genome | Read length | Number of mapped genes | Mapped % |
|---|---|---|---|---|---|
| IR[a] | 51,519,438 | 45,416,972 | 100 | 28,256 | 95.61 |
| ID[b] | 53,444,250 | 44,573,700 | 100 | 33,976 | 93.01 |

[a] IR, iron-rich.

[b] ID, iron-deficient.

This table represents the depth and length of the experimental groups, the accuracy as well.
