# Supplementary material for: Nitric oxide maintains cell survival of Trichomonas vaginalis upon iron depletion
Source: Parasit Vectors. 2015 Jul 25;8:393. doi: 10.1186/s13071-015-1000-5 (PMC4513698; doi:10.1186/s13071-015-1000-5)
Supplement: Additional file 4: — Iron-dependent expression of antioxidants in T. vaginalis . The expression levels of antioxidative defense systems in cells cultured under different iron concentrations were determined by using quantitative RT-PCR. IR, iron rich (80 μM FAC); ID, iron deficiency (180 μM DIP). SOD, superoxide dismutase; Rbr, rubrerythrin; TrxP, thioredoxin peroxidase. [file 13071_2015_1000_MOESM4_ESM.pdf]

Additional file 4. Iron-dependent expression of antioxidants in *T. vaginalis*.

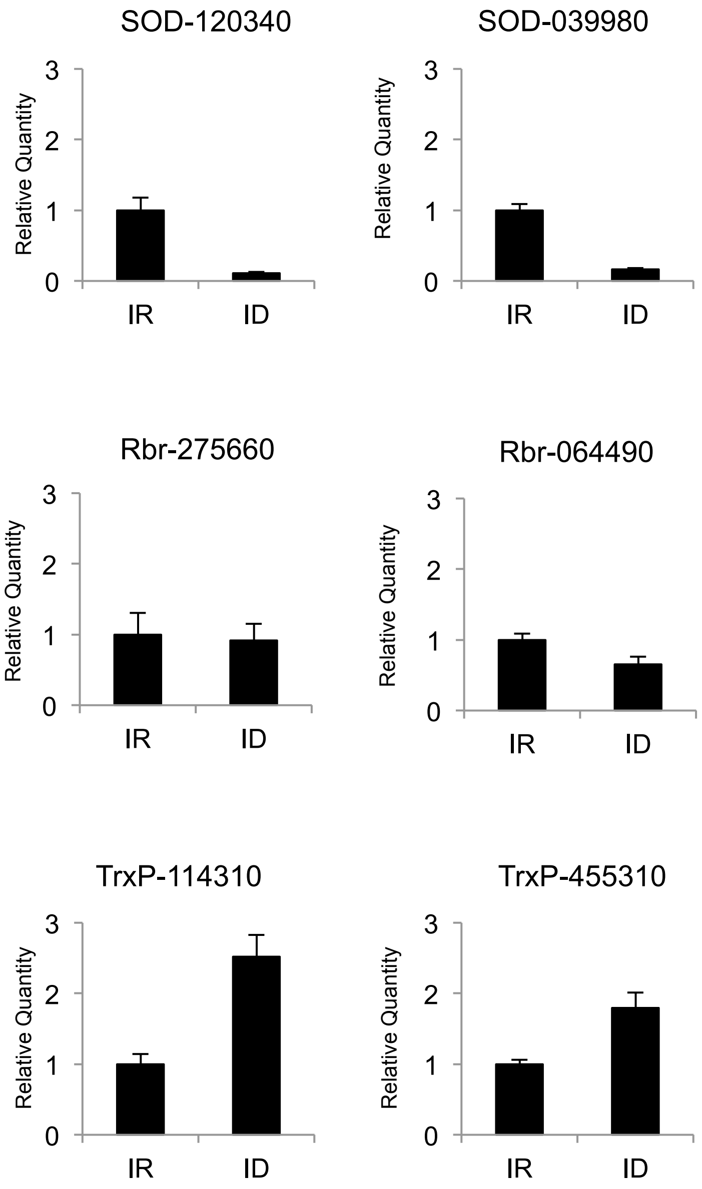

The expression levels of antioxidants in cells cultured under different iron concentrations were determined by using quantitative RT-PCR. IR, iron rich (80 µM FAC); ID, iron deficiency (180 µM DIP). SOD, superoxide dismutase; Rbr, rubrerythrin; TrxP, thioredoxin peroxidase. The data are compared with ribosomal protein L8 for the final adjustment.
