# Supplementary material for: Nitric oxide maintains cell survival of Trichomonas vaginalis upon iron depletion
Source: Parasit Vectors. 2015 Jul 25;8:393. doi: 10.1186/s13071-015-1000-5 (PMC4513698; doi:10.1186/s13071-015-1000-5)
Supplement: Additional file 5: — Validation of NO-related genes identified from transcriptomics analysis. The expression levels of iron deficiency-induced genes were verified by using quantitative RT-PCR. IR, iron rich (80 μM FAC); ID, iron deficiency (180 μM DIP). CMD, 4-carboxymuconolactone decarboxylase; HCP, hybrid cluster protein or hydroxylamine reductase; RCMNS, regulator of cell morphogenesis and NO signaling. [file 13071_2015_1000_MOESM5_ESM.pdf]

Additional file 5. Validation of NO-related genes identified from transcriptomics analysis.

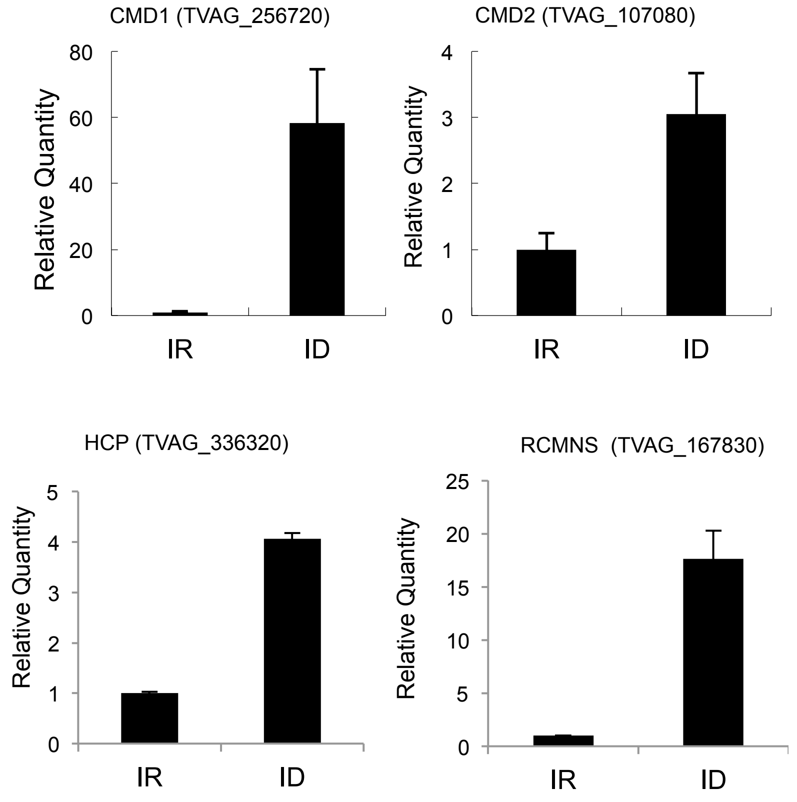

The expression levels of iron deficiency-induced genes were verified by using quantitative RT-PCR. IR, iron rich (80 μM FAC); ID, iron deficiency (180 μM DIP). CMD, 4-carboxymuconolactone decarboxylase; HCP, hybrid cluster protein or hydroxylamine reductase; RCMNS, regulator of cell morphogenesis and NO signaling.
