# Supplementary material for: Nitric oxide maintains cell survival of Trichomonas vaginalis upon iron depletion
Source: Parasit Vectors. 2015 Jul 25;8:393. doi: 10.1186/s13071-015-1000-5 (PMC4513698; doi:10.1186/s13071-015-1000-5)
Supplement: Additional file 6: — NO levels in iron-deficient T. vaginalis treated with NOS and proteasome inhibitors. NO levels in iron-deficient cells treated with the NOS inhibitor (L-NMMA, 3 mM) and the proteasome inhibitor (MG132, 10 μM) for 12 h. [file 13071_2015_1000_MOESM6_ESM.pdf]

Additional file 6. NO levels in iron-deficient *T. vaginalis* treated with NOS and proteasome inhibitors.

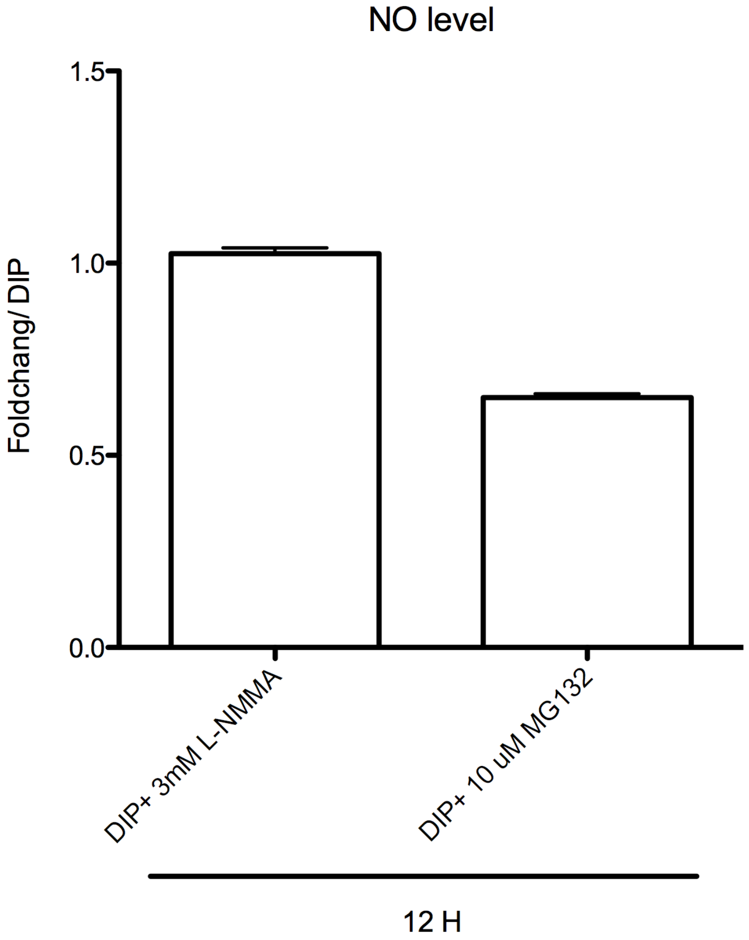

NO levels in iron-deficient cells treated with the NOS inhibitor (L-NMMA, 3 mM) and the proteasome inhibitor (MG132, 10 μM) for 12 hours.
